# Platelet-derived growth factor B induces senescence and transformation in normal human fibroblasts

**DOI:** 10.18632/aging.100577

**Published:** 2013-07-15

**Authors:** David Vindrieux, Baptiste Gras, Merce Garcia-Belinchon, Samia Mourah, Céleste Lebbe, Arnaud Augert, David Bernard

**Affiliations:** ^1^ Inserm U1052, Centre de Recherche en Cancérologie de Lyon, Lyon, F-69000, France; ^2^ CNRS UMR5286, Lyon, F-69000, France; ^3^ Centre Léon Bérard, Lyon, F-69000, France; ^4^ Université de Lyon, Lyon, F-69000, France; ^5^ Cell Death, Senescence, and Survival Group, Departament de Bioquimica i Biologia Molecular and Institut de Neurociencies, Facultat de Medicina, Universitat Autonoma de Barcelona, Barcelona, 08193, Spain; ^6^ INSERM UMR-S940, Laboratoire de Pharmaco-Génétique AP-HP, Hôpital Saint-Louis, Paris, F-75000, France; ^7^ Department of Dermatology AP-HP, Hôpital Saint-Louis, Université Paris Diderot, Sorbonne Paris Cité, F-75000, France

**Keywords:** senescence, transformation, PDGFB, sarcoma

## Abstract

Normal cells enter a senescent state upon aberrant oncogenic signals and this response inhibits tumor initiation and progression. It is now well admitted that intracellular and membrane localized oncogenes can illicit oncogene induced senescence. However, the effect of mitogenic growth factor on cellular senescence is so far largely unknown. Here we show that normal human dermal fibroblasts display a complex response to Platelet derived growth factor B (PDGFB) expression. Indeed, PDGFB expression induces, in the same cell population, both senescence and cellular transformation. Remarkably both populations are sustained with passages suggesting that transformed cells eventually enter a senescent state. This senescence state is p53 dependent as inhibiting the p53 pathway blocks the ability of PDGFB to induce senescence and results in strong cellular transformation increase upon PDGFB expression. The relevance of these observations is supported by the fact that human dermatofibrosarcoma protuberans, skin tumors arising from constitutive PDGFB production with little aggressiveness, also display some senescence hallmarks. Together these data support the view that PDGFB, a mitogenic growth factor, has a limited ability to induce senescence. We propose that this low level of senescence might decrease the transforming ability of this factor without totally abolishing it.

## INTRODUCTION

Tissue homeostasis is dependent upon cellular responses that cells will engage following various detrimental signals. Deregulation or loss of function of these responses leads to the accumulation of damages that might results in disease development. Oncogenic stresses are such detrimental signals that, if not properly recognized, cause cancer development [1]. As a failsafe program in response to an oncogenic stress, cell will enter in a stable form of cell cycle arrest termed senescence. Senescent cells acquire some characteristics such as the secretion of numerous factors that reinforce the cell cycle arrest [2, 3] and allow the detection and elimination of the senescent “damaged” cells by the immune system [4, 5].

Oncogenic stress induced senescence has initially been described in response to oncogenic Ras [6] in normal human fibroblasts. After initial skepticism about the in vivo relevance of these observations, definitive proofs demonstrate the existence of oncogene induced senescence in vivo [7]. Cellular components of the pro- or anti-tumoral circuits are largely investigated for their ability to induce senescence. For example, numerous oncogenes when gained including, but not limited to, RAS, RAF, MEK, AKT, MYC, STAT5 are able to induce oncogene induced senescence [8]. Inversely, loss of tumor suppressor genes such as VHL, PTEN or NF1, results in increase oncogenic signaling and oncogene induced senescence [8]. Less commonly described, the gain of oncogenic receptors such as EGFR or HER2 are also reported to induce oncogene induced senescence [8, 9].

As far as we know, the ability of oncogenic growth factors to regulate oncogene induced senescence is currently unknown. For example, Platelet-derived growth factor B (PDGFB), which is a known oncogene able to transform NIH 3T3 cells [10] and is amplified in some human cancers |^11^, ^12^] has never been tested for its ability to activate oncogene induced senescence failsafe program in normal human cells. In this report, we have investigated the ability of the PDGFB growth factor to regulate oncogene induced senescence in normal human dermal fibroblasts. Surprisingly, we found that PDGFB induces both oncogene induced senescence and transformation in fibroblasts. Oncogene induced senescence phenotype is conserved during cell passages and is also observed in human Dermato-FibroSarcoma Protuberans samples, a tumor relying on PDGFB and displaying limited aggressiveness [11]. PDGFB induced oncogene induced senescence might thus be an intrinsic PDGFB self limiting response opposing the PDGFB induced transformation response, both coexisting.

## RESULTS & DISCUSSION

### PDGFB constitutive expression induces both senescence and transformation in normal human diploid fibroblasts

**Figure 1:**
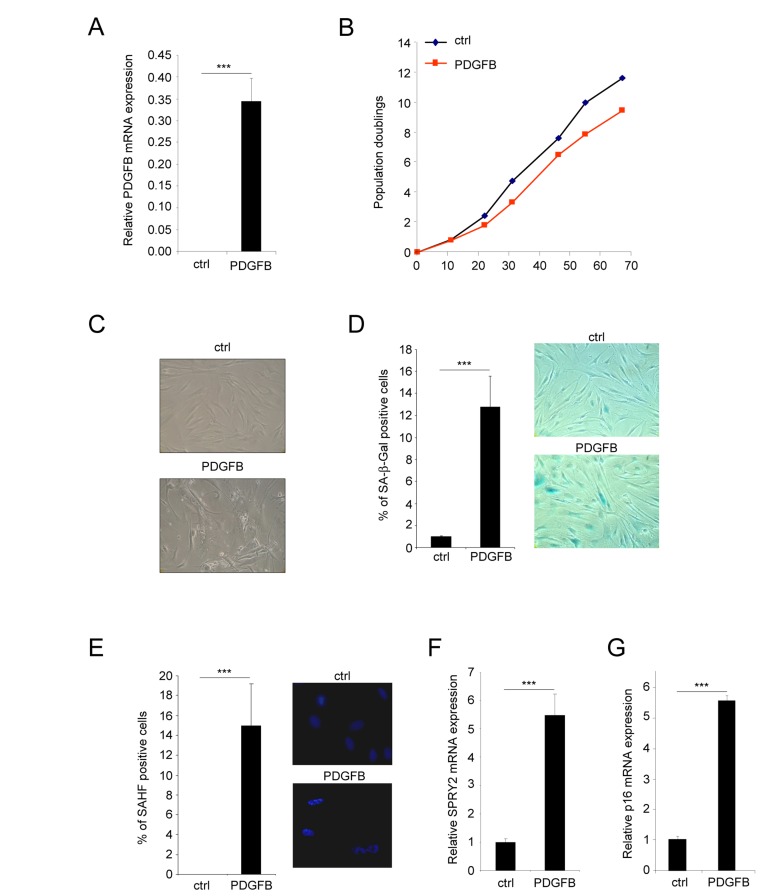
PDGFB induces cellular senescence Normal human dermal fibroblasts were infected with a ctrl or PDGFB-encoding retroviral vectors. **(A)** RNAs were prepared and reverse-transcribed. Quantitative PCR analysis was performed using the primers described in the supplementary Table. PDGFB mRNA levels were analyzed in control cells and in PDGFB-infected cells and normalized to ACTB (Actin-β) levels. Histograms show the mean±SD of a representative experiment from three independent experiments. All experiments were performed in triplicate. **(B)** At the indicated time, 300.000 cells were seeded back in one 10 cm diameter dish for each condition. Population doublings were calculated according to the formula: PD=ln(number of collected cells/number of plated cells)/ln2. **(C)** Seventy five thousand cells from each condition were seeded in a well of 6-well plate and cultured during 7 days. Photographs were taken with a phase contrast microscope. **(D)** Thirty thousand ctrl or PDGFB-expressing cells were seeded in a well of a 12-well plate, and analyzed for their SA-β-Gal activity. The percentage of positive cells in each condition was calculated. A representative experiment from three independent experiments is shown. The histograms represent the mean±SD of a triplicate. To illustrate representative pictures from each condition were shown. **(E)** For the Senescence Associated Heterochromatin Foci (SAHF) analysis, 30.000 cells from indicated conditions were seeded in a well of a 12-well plate and fixed 2 days later with PFA 4%. Nuclei were stained by Hoechst (Sigma). Percentages of SAHF positive cells were calculated and pictures displayed. Histograms represent the mean±SD of a triplicate in a representative experiment from three independent experiments. **(F-G)** RNAs from the indicated cells were prepared and reverse-transcribed. Quantitative PCRs were performed against SPRY2 or p16 and the results were normalized against ACTB using primers described in the supplementary Table. Histogramms represent the mean±SD. Three different experiments were performed and one representative is shown. Statistics were performed using a t-test. *** indicated *p* value<0.005.

To investigate if PDGFB oncogenic growth factor might impact oncogene induced senescence in normal human dermal fibroblasts, we constitutively expressed it. To this end, we have infected fibroblasts with a control or a PDGFB encoding retroviral vector and validated its constitutive expression (Figure 1A). Its constitutive expression resulted in a slight decrease in the growth of these cells when compared to their control counterparts (Figure 1B), suggesting that the PDGFB expression does not induce oncogene induced senescence. Nevertheless, a microscopic analysis enabled us to distinguish two distinct cell populations with striking morphology dissimilarities.

The first population of PDGFB expressing cells displayed features of senescence cells. Indeed they displayed a flattened morphology (Figure 1C), an increase Senescence Associated β Galactosidase (SA-β-Gal) activity (Figure 1D) and an increase Senescence Associated Heterochromatin Foci (SAHF) (Figure 1E). They also displayed an increase expression of Sprouty2 (Figure 1F) and p16 (Figure 1G) mRNA, 2 genes whose expression increased in senescent cells.

**Figure 2:**
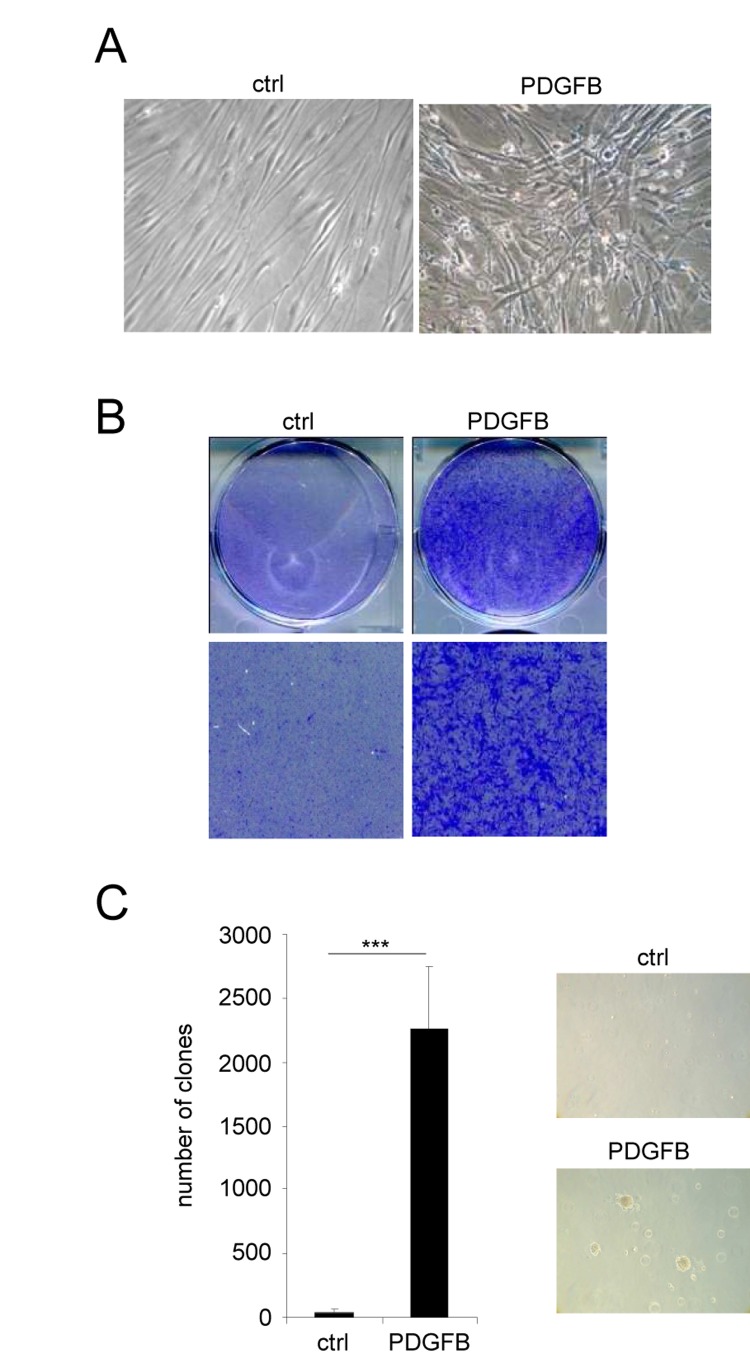
PDGFB induces cellular transformation Normal human dermal fibroblasts were infected with a ctrl or a PDGFB-encoding retroviral vectors. **(A)** Seventy five thousand cells were seeded in a well of 6-well plate and cultured during 7 days. Photographs were taken with a phase contrast microscope. **(B)** The cells were seeded at high densities. Two weeks later they were PFA-fixed and stained with 0.05% crystal violet (Sigma-Aldrich). **(C)** Soft agar experiments were performed by seeding 50.000 cells per well in 6-well plates. Plates were incubated for 4 weeks and colonies were counted. Histograms represent the mean±SD. Three different experiments were performed and one representative is shown. Statistics were performed using a t-test. *** indicated *p* value<0.005.

The second population of PDGFB expressing cells also displayed an altered morphology when compared to control cells. However, they did not display any characteristics of senescent cells but instead these cells had lost their contact inhibition: a feature of transformed cells (Figure 2A). Cellular transformation was further supported by the ability of PDGFB expressing cells to form colonies whereas the control cells stopped growing when reaching confluence (Figure 2B). Furthermore, PDGFB expressing cells were found to grow and to form colonies in soft agar when compared to control cells (Figure 2C). Together, these data show that PDGFB constitutive expression results in 2 distinct and “opposite” phenotypes in fibroblasts.

Some cellular oncogenes have been shown to induce both senescence and transformation, but theses processes generally appeared in a sequential manner; a mitogenic effect during the first days followed by senescence activation and subsequent proliferation arrest [13]. Interestingly, here, these 2 populations were not seen in a sequential manner but instead were triggered simultaneously a few days after infection. Importantly, these 2 populations are maintained with passages explaining the slow growth rate of the PDGFB expressing cells (Figure 1B). To maintain both populations, senescent cells have to be generated by the transformed cells during passages suggesting that PDGFB induced transformation is subject to stochastic events that have a tendency to disturb the homeostasis of the transformed cells, and then leading to senescence. In the meantime, the PDGFB induced senescent population might exert an intrinsic inhibition towards the PDGFB induced transformed population explaining that the transformed cells do not out grow.

### Favoring an escape from PDGFB induced senescence increases PDGFB induced transformation

**Figure 3:**
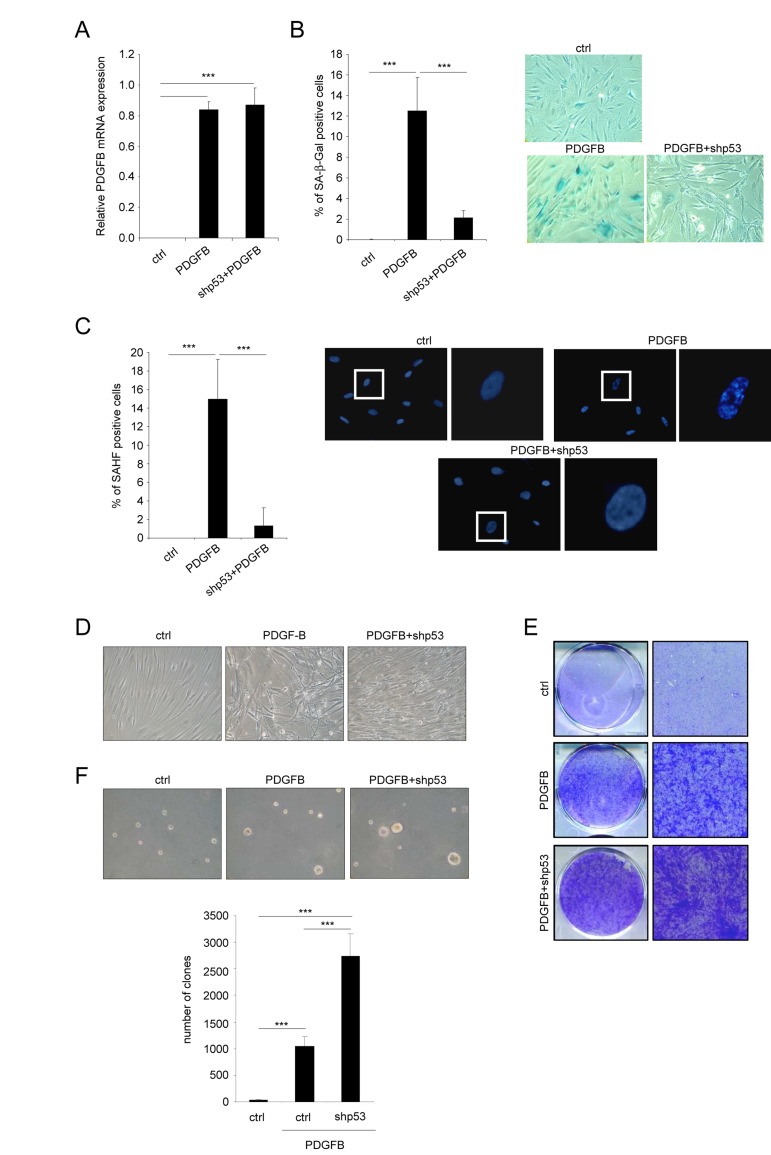
Favoring an escape from PDGFB-induced senescence increases PDGFB-induced transformation Fibroblasts were infected with a ctrl, a PDGFB- with or without an shRNA against p53 encoding vector. **(A)** RNAs were extracted and reverse-transcribed. Quantitative PCRs were performed against PDGFB and the expression normalized with ACTB. **(B)** Twenty five thousand cells were seeded in a well of 12-well plate, and analyzed for their SA-β-Gal activity 3 days later. **(C)** Cells were seeded as indicated in (B) and fixed 2 days later with PFA 4%. After DNA staining by Hoechst, percentage of SAHF-positive cells was calculated. **(D)** Seventy five thousand cells were seeded in a well of 6-well plate and cultured during 7 days. Representative photographs are presented. **(E)** Indicated cells were seeded at high densities. Two weeks later they were PFA-fixed and stained with crystal violet. **(F)** Fifty thousand cells were plated in agar in 6-well plates. After 3 weeks colonies were quantified and photographed. All histograms shown in this figure represent the mean±SD. Three different experiments were performed and one representative is shown. t-test was used for statistical analysis. *** indicated *p* value < 0.005.

We next wanted to know whether the senescent population limits the ability of PDGFB to transform the cells. To this end, we wished to eliminate the PDGFB induced senescent population by decreasing p53 expression, a key player of the senescence response [6, 14]. We then ectopically expressed PDGFB with or without an shRNA targeting the p53 protein (Figure 3A). As expected, p53 knockdown strongly decreased the appearance of the senescence population in the PDGFB expressing cells as measured by loss of the SA-β-Gal positive cells (Figure 3B) and the decreased SAHF positive cells (Figure 3C). As hypothesized, loss of the senescent population in p53 knockdown cells allowed the transformed population to increase as seen by the increase cells with the transformed morphology (Figure 3D), the increase number of cells forming colonies and loosing the contact inhibition (Figure 3E), and the increase ability to form clones in soft agar (Figure 3F). These results support the view that the low senescence response induced by the PDGFB limits the growth of the PDGFB transformed cells.

### Senescence markers are observed in PDGFB dependent human tumor samples

**Figure 4:**
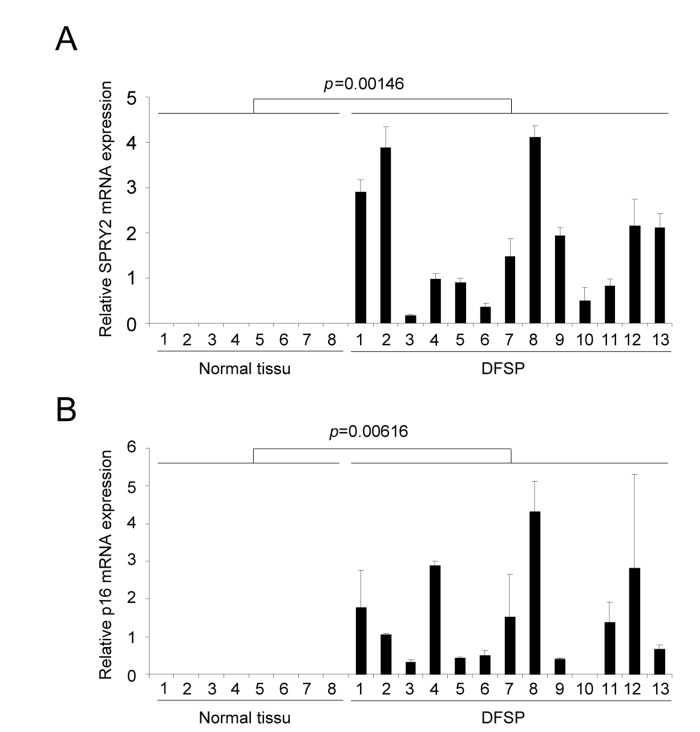
DFSP displays accumulation of senescence markers RNAs were prepared from normal skin or from DFSP samples. They were next reverse-transcribed and qPCR were performed against **(A)** SPRY2 and **(B)** p16 and the results were normalized against actin. P value was calculated using a t-test.

Dermatofibrosarcoma protuberans (DFSP) is a rare and locally aggressive skin sarcoma that appears at different age, starting at childhood. This type of tumor can grow and form large lesion, but it barely forms metastasis. DFSP displays in most if not all cases a chromosomal rearrangement resulting in the production of COL1A1/PDGFB precursor chimera [15]. The COL1A1/PDGFB precursor is next processed to the oncoprotein PDGFB, which is thought to be at the origin of DFSP [11]. Because our in vitro results suggest that PDGFB triggers senescence, we decided to investigate whether these human PDGFB depending tumors, with limited aggressiveness, might display some senescence features. The two senescence markers examined, Sprouty2 (Figure 4A) and p16 (Figure 4B), were found strongly up regulated in DFSP when compared to normal tissues. This supports the view that an increase of PDGFB signaling might result in the appearance of tumors composed of transformed and senescent cells, limiting the intrinsic aggressiveness of the tumors. Interestingly, the very rare aggressive version of the DFSP, the DFSP Fibrosarcomatous, may displays p53 loss of function [16]. This is in agreement with the idea that p53 loss might decrease the ability of PDGFB to induce senescence and potentially DFSP aggressiveness.

Our results also point out that a single oncogene such as the PDGFB, which according to our data is a weak senescence inducer, might trigger transformation with a minimal number of genetic events in fibroblasts. This result is rather unexpected at first sight. Indeed, classical cellular oncogenes such as the ones in the RAS-RAF-MEK pathway, that are strong oncogene induced senescence inducer, need to cooperate with other genetic events, such as loss of function of the p53 and Rb pathways, to transform normal human cells [17, 18]. In this study, we thus describe that the PDGFB oncogenic growth factor is able to activate cellular transformation of primary fibroblasts. Nevertheless, low level of senescence is a self limiting response that decreases the transformed phenotype. The price to pay for PDGF induced transformation, in a context of limited genetic events, is that although the transformed cell population grows, this growth is slow, probably limited by the effects of neighboring senescent cells.

## METHODS

### Cell Culture

Human Dermal Fibroblast (Lonza) and virus producing GP293 cells (Clontech) were cultured in DMEM (Invitrogen). All medium were supplemented with 10% FBS (Lonza), 1% penicillin/streptomycin (Invitrogen), 0.36% gentamycin (Invitrogen).

### Vectors

pBABE/PDGFB and empty pBABE were provided from Addgene and were described in [19]. pSR/shp53 retroviral vector encoding an shRNA directed against p53 was described in [20].

### Transfection and infection

GP293 cells were transfected by PEI reagents according to the manufacturer's recommendations (Euromedex). Two days after transfection, the viral supernatant mixed with fresh medium (1/2) and polybrene (final concentration: 8 μg/mL) was used to infect target cells. HDF cells were infected for a period of 7 hours. Importantly, the infection protocols were designed so that practically all cells were infected, as judged by the results of infection with a GFP control. One day post-infection, cells were eventually selected with puromycin at the final concentration of 250 ng/ml.

### RNA extraction, retro-transcription and PCR

Total RNA extraction was performed using a phenol–chloroform method using TriReagent (Sigma-Aldrich, Saint Louis, MO, USA). The synthesis of cDNA was performed from 3 μg of total RNA using the First-Strand cDNA Synthesis Kit (GE Healthcare, Chalfont St Giles, UK). The RT reaction was diluted 1/60 and used as cDNA template for qPCR analysis. TaqMan quantitative PCR analysis was carried out on a LightCycler 2.0 System (Roche Applied Science). PCR mixtures contained LightCycler TaqMan mix, 200nM primers and 1.67μl of cDNA template in a 6.67μl reaction volume. Housekeeping gene ACTB was used for normalization of PDGF-B, p16/INK4a and SPRY2 mRNA expression in each sample type. Real-time PCR intron-spanning assays were designed using the ProbeFinder software (Roche Applied Science).

### Colony formation assays

Colony formation assays were carried out in 6 or 12-well plates. Thirty thousand or 60.000 HDF cells were seeded in 6 or 12-well plates respectively. Ten to forteen days after seeding, the cells were washed with PBS, fixed with 4% paraformaldehyde, and stained with 0.05 % crystal violet (Sigma-Aldrich).

### SA-β-Gal analysis

SA-β-Gal analyses were performed as in [21]. At least 5 different fields were counted for each condition representing at least 500 events.

### Senescence-associated heterochromatin foci (SAHF) staining

For senescence-associated heterochromatin foci analysis [22], cells were fixed with formaldehyde 4 %, wash with PBS and stained with Hoechst (Sigma). DNA staining was examined under a Zeiss fluorescence microscope.

### Soft-agar assays

To measure anchorage-independent growth, cells were detached with trypsin and re-suspended in growth medium. Base agar was prepared with 0.75 % low-melting agarose (Lonza) in growth medium. The top agar contained the suspension of cells in 0.45% low-melting agarose. Plates were incubated for 4 weeks at 37°C and colonies were counted under a bright light microscope.
